# Weight Loss Supplements

**DOI:** 10.3390/molecules28145357

**Published:** 2023-07-12

**Authors:** Irene Dini, Andrea Mancusi

**Affiliations:** 1 Department of Pharmacy, University of Naples Federico II, Via Domenico Montesano 49, 80131 Napoli, Italy; 2 Department of Food Microbiology, Istituto Zooprofilattico Sperimentale del Mezzogiorno, Via Salute 2, 80055 Portici, Italy

**Keywords:** antiobesity, food-derived moieties, antiobesity phytochemicals, prebiotics, microbial-derived moieties, probiotics, metabiotic, parabiotic, postbiotic

## Abstract

Being overweight or obese can predispose people to chronic diseases and metabolic disorders such as cardiovascular illnesses, diabetes, Alzheimer’s disease, and cancer, which are costly public health problems and leading causes of mortality worldwide. Many people hope to solve this problem by using food supplements, as they can be self-prescribed, contain molecules of natural origin considered to be incapable of causing damage to health, and the only sacrifice they require is economic. The market offers supplements containing food plant-derived molecules (e.g., primary and secondary metabolites, vitamins, and fibers), microbes (probiotics), and microbial-derived fractions (postbiotics). They can control lipid and carbohydrate metabolism, reduce appetite (interacting with the central nervous system) and adipogenesis, influence intestinal microbiota activity, and increase energy expenditure. Unfortunately, the copious choice of products and different legislation on food supplements worldwide can confuse consumers. This review summarizes the activity and toxicity of dietary supplements for weight control to clarify their potentiality and adverse reactions. A lack of research regarding commercially available supplements has been noted. Supplements containing postbiotic moieties are of particular interest. They are easier to store and transport and are safe even for people with a deficient immune system.

## 1. Introduction

The World Health Organization has estimated that 24% of the world’s population will be obese by 2030 [1]. Obesity is a pathology due to an altered energy balance between the intake and consumption of calories [1]. The development of obesity is linked to sociological, psychological, evolutionary, biological, institutional, and economic aspects [2,3,4].

According to the latest annual statistics in the FAO (Food and Agriculture Organization) report, people in North America and Europe consumed 3540 calories per day in 2021 instead of the 2000 calories per day necessary for women and 2500 for men [5]. Adverse social factors (e.g., poor life quality), health problems, and mortality are related to obesity [6,7]. Adults living with obesity in their 20s have a reduced life expectancy of 5.6 to 10.3 years [8]. In 2019, five million premature deaths were ascribable to being overweight or obese [9]. Cancer, type 2 diabetes, and cardiovascular and chronic kidney disease are responsible for deaths linked to obesity [10]. Moreover, obesity impacts quality of life, mental health, and sexual function [11]. An enhanced meal calorie intake due to the possibility of eating highly caloric and palatable food that can produce dependences [12,13], an altered metabolite metabolism due to fast meals, and a sedentary lifestyle are the leading causes of obesity [14]. Genetic components regulate an individual’s response to the accumulation of excessive energy in their body’s fat stores [15]. In some people, fat collects predominantly in the abdominal adipose tissue and infiltrates other visceral organs, promoting cardiometabolic risk. Adipose tissue is an active endocrine and paracrine organ that secretes molecules (e.g., adipokines, hormones, and inflammatory cytokines) [16] that can control the immune response, energy homeostasis, and inflammation. In obesity, adipose tissue activates the proinflammatory cascade, systemic insulin resistance, fatty acid, and glucose dysregulation. This dysregulation damages the arteries, heart, skeletal muscle, liver, and pancreas and causes metabolic, hormonal, and target-organ alterations in the function of the body weight’s magnitude and its distribution [17]. Restricted calorie regimens and physical exercise decrease the risk of obesity, but these approaches are not rapid and require substantial patient compliance [18]. It is possible to limit obesity pharmacologically (e.g., by using fenfluramine, orlistat, coreaserin, cetlistat, rimonabant, sibutramine, phentermine, and topiramate) [19,20] or by taking food supplements. The latter approach is chosen by those who are afraid of the side effects produced by drugs, are attentive to maintaining a healthy and eco-sustainable lifestyle, and hope that natural molecules do not cause damage to their health. This review summarizes the weight management supplements on the market. Their mechanisms of action, side effects, and sector perspectives are discussed.

## 2. Search Methodology

Scopus, SciFinder, and Google Scholar were employed to search the research papers and reviews on body weight control dietary supplements published since 1979. Patents were searched on Google Patents. The keywords/phrases/sentences used to search the scientific works or patents related to body weight control dietary supplements were as follows: obesity supplement control, dietary supplement obesity, dietary supplement obesity patent, weight loss control supplement, and appetite suppressing supplement. Topics, editorials, conference proceedings, and commentaries were not considered.

## 3. Obesity

**Figure 1:**
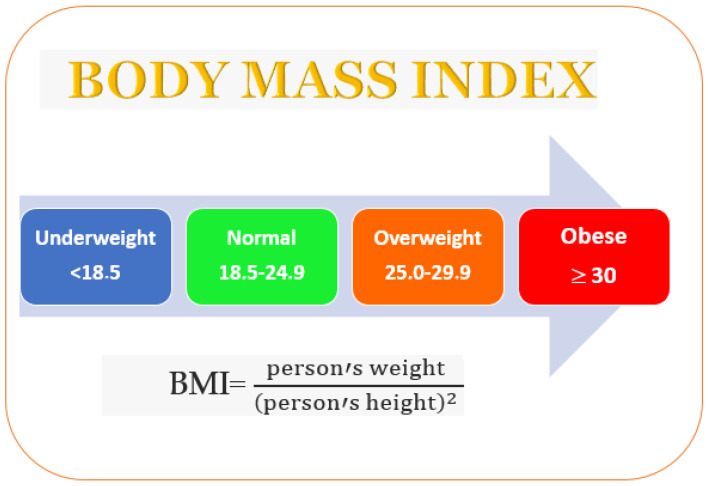
Overweight incidence evaluated by the body mass index.

The body mass index (BMI) values body fat based on a person’s weight and height. A person whose BMI is over 25 is considered to be overweight, and obese if it is over 30 (Figure 1). Family genetics (a propensity to accumulate fat), psychological factors, and lifestyle (poor exercise or dietary habits) can result in obesity [21]. In living organisms, lipids and fatty acids are formed from glucose. Successively, fatty acids are esterified into triglycerides and stored in adipose tissue. Amylases and glucosidases are the key enzymes that metabolize carbohydrates into glucose [22]. Increased glucose levels determine the insulin release from pancreatic cells and induce glycogenesis, glycolysis, and lipogenesis [23].

Pancreatic lipase is a critical enzyme in dietary fat digestion. It reduces the fat deposition into adipose tissue and controls the digestion and absorption of triglycerides [24]. This lipase is upregulated by glucagon and epinephrine and downregulated by insulin [25]. Adipose tissue regulates obesity. Adipocytes act as energy storage, detect energy demands, and produce paracrine factors to regulate other metabolic tissues. In obesity, adipose tissue becomes severely dysfunctional, does not store excess energy, causes ectopic fat deposition [26], enhances the levels of free fatty acid metabolites (e.g., ceramide, long-chain fatty acyl Coenzyme A, and di-acyl glycerol) [27], and regulates insulin resistance by constraining the protein-kinase B (PKB) pathway [28].

**Figure 2:**
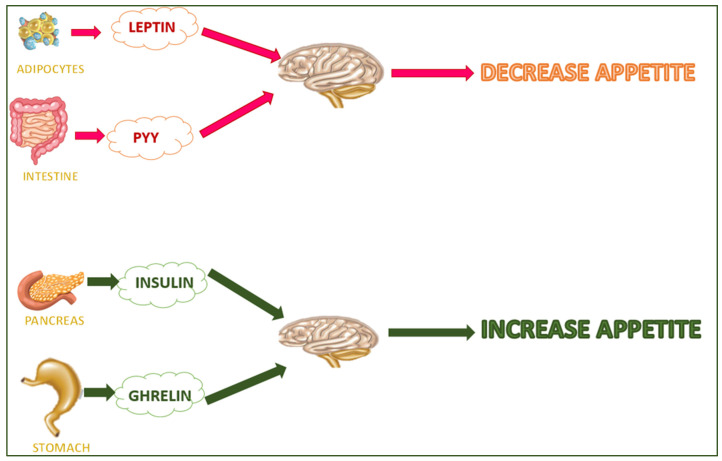
Hunger/satiety-regulating hormones.

Hyperinsulinemia increases the ATP level and downregulates the AMP-activated protein kinase (AMPK) pathway [29]. In obesity, preadipocyte differentiation into mature adipocytes is promoted [30], as is the production of inflammatory cytokines (such as the Tumor necrosis factor alpha (TNF-α) and some interleukins such as IL-6, IL-1, and IL-18) [31]. TNF-α downregulates insulin sensitivity (improving IκB kinase/NF-κB signaling), glucose uptake (preventing the GLUT-4 transporter), the 5′ AMP-activated protein kinase (AMPK) pathway, lipogenesis (reducing PPARγ expression), and increases lipolysis [32]. Some hormones (e.g., leptin, insulin, adiponectin, and ghrelin) are involved in the etiopathogenesis of obesity. Leptin is released by white adipose tissue (WAT) and regulates the brain–gut axis. It controls appetite and metabolism by impeding the synthesis and release of neuropeptide Y in the arcuate nucleus. The leptin isoform b (LEP-Rb) regulates the energy balance and body mass in the ventromedial hypothalamic nucleus, arcuate nucleus, lateral hypothalamic nuclei, and dorsomedial hypothalamic nucleus and decreases appetite [33]. Insulin (secreted from pancreatic beta cells) converts signals to the brain and decreases food intake (over the long term) and rapid energy outflow. Brain insulin signaling regulates systemic and organ-specific metabolism, often in a complementary manner [34] (Figure 2). Signals from leptin and insulin communicate to reduce food and energy intake [35], the metabolisms of carbohydrates and lipids [36], fatty acid oxidation, and glucose uptake in the skeletal muscle and liver [37]. Adiponectin can activate the adenosine monophosphate-activated protein kinase (AMPK) and decrease acetyl CoA carboxylase and malonyl CoA activities [38,39].

Adiponectin is secreted from adipose tissue and controls energy homeostasis and the metabolisms of carbohydrates and lipids [36]. It improves the fatty acid oxidation, hepatic insulin activity, and glucose uptake in the skeletal muscle and liver [37]. Adiponectin can activate the adenosine monophosphate-activated protein kinase (AMPK) and decrease acetyl CoA carboxylase and malonyl CoA activities [38,39]. The stomach secretes ghrelin (the hunger hormone), which stimulates food intake and adiposity [40]. Finally, endoplasmic reticulum stress can affect insulin resistance, activating the Jun N-terminal kinase (JNK) and inhibitory kappa B kinase (IKK) pathways [41].

## 4. Supplement Regulation

Urbanization and income growth worldwide have increased the demand for products that control weight management. This segment is expected to grow significantly in the coming period due to the prevalence of obesity among adults and children worldwide linked to changing food habits [42]. The global dietary supplements market will probably reach 327.4 billion USD by 2030. Dietary supplements are regulated differently around the world. In the USA, they are regulated as food by the FDA (Food and Drug Administration) under the DSHEA of 1994 (Dietary Supplement Health and Education Act) [43]. In the United Kingdom, food supplements are regulated by the Department of Health and Social Care (England), Food Standards Scotland (Scotland), Welsh Government (Wales), and Food Standards Agency (Northern Ireland). They are defined as “food whose purpose is to supplement the normal diet and which is a concentrated source of a vitamin or mineral or other substance with a nutritional or physiological effect, alone or in combination and is sold in dose form” [44]. In other jurisdictions, they are considered to be therapeutic goods, food supplements, prescription medicines, or controlled substances [45]. In Italy, the Directive 2002/46/EC and Legislative Decree 21 May 2004 n. 169 regulate dietary supplements as “food products that can supplement the common diet. They are a source concentrate of nutrients, such as vitamins and minerals, or other substances having an effect nutritional or physiological, in particular—but not exclusively—amino acids, essential fatty acids, probiotic microorganisms, fibers, and extracts of vegetable origin, both mono-compound and multi-compound” [46]. The uneven legislation on the marketing of these products around the world can confuse consumers. It is hoped that convergence on this matter can be achieved as soon as possible.

## 5. Weight Management Supplements

**Figure 3:**
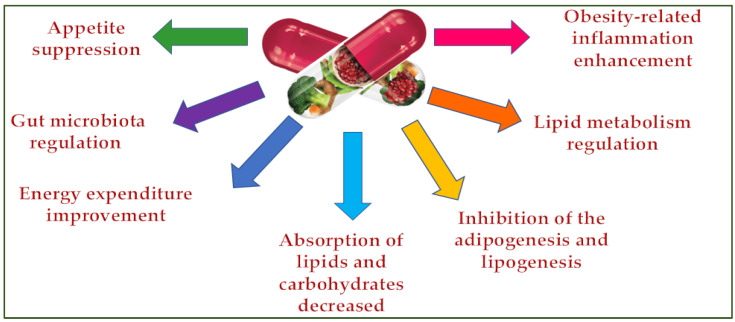
Dietary supplements’ antiobesity action mechanisms.

Dietary supplements can control being overweight by inhibiting the appetite [47], lipid and carbohydrate absorption [48], adipogenesis and lipogenesis [49], regulating lipid metabolism and the gut microbiota [50], and improving energy consumption [51] and obesity-related inflammation (Figure 3) [52].

### 5.1. Plants Extract in Supplements for Weight Control Management

Usually, weight loss supplements are multi-ingredient preparations (an average of 10 ingredients are enclosed) [53]. It is difficult to determine their effects on the body due to the recipes’ complexity and different dosages, extract types, and administration times used in studies. Some food or medicinal plants are employed in weight control treatments. Their effects are mainly linked to secondary metabolites (e.g., polyphenols and saponins, etc.) [54,55], unsaturated fatty acids, and fibers. Natural products that are in used in weight control management include green tea, garcinia cambogia, turmeric, ginger, coffee, chili pepper, spirulina, licorice, hibiscus sabdariffa, white bean, and yerba maté, etc.

Green tea (GT) extract decreases waist circumference (WMD: −2.06 cm) when GT of ≥800 mg/day for <12 weeks or GT of <500 mg/day for 12 weeks is consumed [56]. The consumption of green tea extract for up 14 weeks decreases body weight (BW: 1.8 kg) and body mass index (BMI: 0.65 kg/m^2^) [56]. Unfortunately, some studies have reported that green tea extract can cause liver damage [57,58]. Dexaprine (a multi-ingredient supplement with green tea extract) has caused some consumers emesis, anxiety, and tachycardia [59]. The Linea Detox (with green tea extract) has produced anaphylactic reactions [60].

A meta-analysis of 2011 subjects showed that garcinia cambogia supplementation can cause BW loss (−0.88 kg) without affecting BMI [61]. Another meta-analysis of 2020 subjects showed that Garcinia cambogia supplementation for 8–12 weeks reduced BW (−1.34 kg), fat mass (−0.42%), BMI (0.99 kg/m^2^), and waist circumference (WC−4.16 cm) [62].

A meta-analysis of 876 subjects (53% women) showed that curcumin supplementation (≥1000 mg/d) with a treatment duration of ≥8 weeks decreased BW (−1.14 kg) and BMI (−0.48 kg/m^2^) [63]. Some multi-ingredient supplements with garcinia cambogia extract have showed toxic effects. The hydroxycut produces liver damage, heart arrhythmia, death [64,65,66], thermatrim, and leukoencephalopathy [67].

A meta-analysis of 470 subjects demonstrated that ginger intake (with doses ranging from 200 to 3000 mg/day with a duration of treatment ranging between 2 and 12 weeks) reduced BW (−0.66 Kg), waist-to-hip ratio (−0.49), hip ratio (−0.42), fasting glucose (−0.68 mmols/L), and insulin resistance index (−1.67), and increased HDL-cholesterol (+0.40 mg/dL) but did not affect insulin, BMI, triglycerides, and total- and LDL-cholesterol levels [68]. A meta-analysis of 695 subjects demonstrated that green coffee intake for 4 and 8 weeks reduced BMI by −0.403 kg/m^2^, with no significant change in BW (−0.585 kg) and WC (−0.847 cm). Short supplementation periods (less than 4 weeks) have no effect [69].

A meta-analysis of 191 participants demonstrated that consuming several doses of capsaicinoids (contained in chilly pepper) daily decreased energy intake [70]. A meta-analysis demonstrated that spirulina supplementation (with doses ranging from 1 to 4.5 g/day with a duration of treatment ranging between 6 and 12 weeks) decreased BW (−1.56 Kg), body fat percentage, and waist circumference, but no changes in BMI and waist-to-hip ratio were observed [71]. The French Agency for Food, Environmental, and Occupational Health & Safety claimed that spirulina has no health risk when up to several grams are used daily [72]. A meta-analysis that evaluated the consumption of licorice or its derivatives (300–900 mg/day with a duration ranging from two to 16 weeks) showed that licorice consumption reduced BW, dependent on the dose and duration of the treatment (−0.433 kg) and the BMI of patients (−0.150 kg/m^2^), and increased diastolic blood pressure (DBP: 1.737 mmHg) [73]. A study on supplementation with lippia citriodora and hibiscus sabdariffa demonstrated that their supplementation reduced the appetite sensation in overweight and obese populations (−3.36 calorie intake after an ad libitum meal) due to variations in hunger-related hormones (leptin −1.07 ng/mL and incretin 1.11 ng/mL) [74]. A meta-analysis of 573 participants that evaluated a *Phaseolus vulgaris* supplementation (3000 mg/day with a duration ranging from 2 to 16 weeks) showed that it reduced BW (−1.08 kg) and body fat (from −2.35 kg to −4.163 kg) [75].

### 5.2. Dietary Supplements Able to Decrease the Appetite

Appetite control can reduce weight gain [76]. Some supplements that can suppress the appetite are reported in Table 1. They can contain grains (e.g., wheat, oats, corn, rice, rye, or barley) [77], prebiotics (e.g., fructosan and inulin) [78], secondary metabolites such as saponins (e.g., pregnane glycosides and stavarosides) [79], methylxanthines (e.g., caffeine, theobromine, and theophylline) [80], and hydrolyzed yeast proteins [81].

### 5.3. Dietary Supplements Able to Interact with the Central Nervous System

Some supplements can promote antiobesogenic effects, interacting with the central nervous system and determining the release of hormones, such as the neuropeptide Y (that can delay satiety and promote food intake), norepinephrine (that can increase lipolysis), the POMC/CART (that can regulate food consumption) [82], the melanocortins and α-melanocyte-stimulating hormone (that can regulate the appetite and are affected by leptin and insulin) [83], and serotonin (that can regulate food intake) (Table 1). The plant secondary metabolites that can interact with the hormones released by the central nervous system are ephedrine (that acts as a sympathomimetic agent) [84], the red ginseng’s saponins (protopanaxadiol and protopanaxatriol type that act by downregulating leptin and neuropeptide Y) [85,86], the garcinia’s hydroxy citric acids (that control the glucose and uptake of serotonin level) [87,88], the amines in citrus with aromatic rings (that improve serotonin levels) [89], and fucoxanthin isolated from brown seaweed (that impacts insulin levels) [90].

### 5.4. Dietary Supplements That Interact with the Hormones in the Digestive System and Adipose Tissue

Some dietary supplements suppress the appetite by regulating the secretion of hormones in the digestive system (e.g., the ghrelin in the stomach) and adipose tissue (e.g., leptin, secreted by adipocytes [91], the AMP-activated protein kinase that controls energy metabolism [92], and the carnitine palmitoyl transferase 1A and cofactor for the beta-oxidation of fatty acids that enhance the fatty acid oxidation) [93]. Some supplements’ patents that are based on plants or secondary metabolites that can interact with the hormones in the digestive system and adipose tissue are reported in Table 1.

### 5.5. Prebiotics in Weight Control Supplements

Prebiotics are non-viable food components (e.g., non-digestible carbohydrates, peptides, proteins, and lipids) [94] that can positively impact beneficial bacteria’s activity (e.g., *Lactobacillus* and *Bifidobacterium*) and/or growth in the gut microbiota [95]. They are not hydrolyzed by gastric acidity and mammalian enzymes. Moreover, prebiotics do not get absorbed into the gastrointestinal tract, are fermented by the gut microbiota, and are beneficial to a host’s health [96]. The prebiotic, non-digestible carbohydrates include resistant starch, non-starch polysaccharides, and oligosaccharides composed of three–nine sugar units [97,98], which endogenous enzymes cannot hydrolyze [99]. By imitating intestinal binding sites, some prebiotics impede the pathogenic microbiota’s adhesion to the gastrointestinal tract [100]. These prebiotics can modulate the immune system by upregulating interleukins and immunoglobulins, downregulating proinflammatory interleukins [101,102], and improving short-chain fatty acids’ (SCFAs) production [103]. The SCFAs improve the intestinal barrier integrity, are an essential indicator of bacterial fermentation in the colon [104], protect against inflammation, regulate mucus production [105], and constrain obesity [106]. Some patents containing prebiotics are reported in Table 1.

### 5.6. Probiotics in Weight Control Supplements

Probiotics are live microorganisms that affect human health when consumed adequately [107]. They control being overweight, enhancing the gut barrier function, decreasing metabolic endotoxemia, systematic inflammation, gut permeability, energy hemostasis, and appetite regulation. They can deconjugate the bile acids interfering with lipid absorption, increase SCFAs, and stimulate intestinal peptide synthesis [108,109,110]. The probiotic *L. rhamnosus* GG strain can constrain obesity via the upregulation of adiponectins [111]. A mix containing *Bifidobacterium*, *Lactococcus*, and *Propionibacterium* showed a significant reduction in the total body and visceral adipose tissue [112]. Some patents containing probiotics are reported in Table 1.

### 5.7. Symbiotics in Weight Control Supplements

Synbiotics are “a mixture comprising live microorganisms and substrate(s) utilized by host microorganisms that confer a health benefit on the host” [113]. The complex mixtures of bacterial strains and different dosages of prebiotic fibers in symbiotics can modulate the metabolic activity in the intestine, upregulate microbiota development, short-chain fatty acid, carbon disulfides, ketones, and methyl acetate concentrations, decrease pathogens, and inactivate nitrosamines and other cancerogenic substances [114]. Moreover, synbiotics can regulate weight by activating a host’s satiety pathways [115] and energy expenditure. Synbiotics containing *Lactobacillus gasseri* strains galactomannan and/or inulin fibers have shown antiobesity effects, improving the SCFA levels and upregulating the microbiota [116]. Some patents containing symbiotics are reported in Table 1.

### 5.8. Postbiotics in Weight Control Supplements

Postbiotics are products (microbial cells or cellular factors that have been attenuated with or without metabolites) or metabolites produced by bacteria or liberated after bacterial lysis, which have a beneficial role in human health [117,118]. Gut bacteria secrete low-molecular-weight metabolites that regulate their growth, promote cell-to-cell communication, and protect against environmental stresses [119,120,121]. The *Lactobacillus*, *Bacillus*, *Bifidobacterium*, *Faecalibacterium*, and *Streptococcus* genera can produce postbiotics [122,123]. These postbiotics emulate probiotics’ actions and have a better shelf-life, easier packaging, and minor transport requirements. SCFA, enzymes, peptides, vitamins, and teichoic acids exemplify postbiotics [124]. Acetate, propionate, and butyrate are the most rapresentative SCFAs [124,125]. Butyrate and propionate can positively downregulate the gut hormones and decrease food intake [126]. Acetate acts as a lipogenic substrate propionate that can moderate lipogenesis by downregulating the fatty acid synthase (in the liver). Therefore, the acetate/propionate ratio is crucial for de novo lipogenesis [127]. Moreover, propionate and butyrate can attenuate body weight and adiposity by improving the expression of gluconeogenesis genes and intestinal gluconeogenesis [128]. Acetate can enhance BAT (brown adipose tissue) thermogenesis and the browning of WAT (white adipose tissue) [129]. Interestingly, it has been found that Bacteroidetes mainly produce acetate and propionate, while Firmicutes produce butyrate [129]. Nevertheless, different phyla or genera are unrelated to producing a specific SCFA. Vanillic acid (a metabolite from anthocyanin metabolism) can enhance BAT thermogenesis and WAT browning [130]. The ketoA (a metabolite from linoleic acid metabolism) can improve energy expenditure [130]. Among the cell wall components, muramyl dipeptide (MDP), S-layer proteins (SLPs), lipoteichoic acid (LTA), and exopolysaccharide (EPS) have shown antiobesity activities [131]. The bacterial cell wall components, peptidoglycans (e.g., diaminopimelic acid (meso-DAP) and muramyl dipeptide (MDP)), can decrease adipose inflammation and glucose intolerance [132,133,134,135]. Surface-layer protein (SLP) glycoproteins [136] can decrease lipid accumulation [137] and enhance adipogenesis, insulin resistance, and systemic inflammation [138]. Lipoteichoic acid (LTA) has immunomodulatory, anti-inflammatory [139,140], and fat-reducing effects (controlling the insulin-like signaling pathway) and regulates lipid metabolism, aging, and immunity [141,142,143]. The glycocalyx exopolysaccharide (EPS) has antioxidant, antitumor, anti-inflammatory, antiviral, immunological modulation, antimicrobial, and anti-biofilm activities [144,145], reduces adipocyte function [146], and inhibits fat deposition and the upregulation of WAT browning by downregulating acetyl-CoA carboxylase (ACC) expression and impeding gluconeogenesis [147]. Cell-free extracts with a high protein content (27.5% crude protein) [148] can control lipid metabolism, increasing browning and lipolysis [149]. Regarding their safety, postbiotics do not determine resistance and have anti-virulence properties [150]. These features are crucial for children with developing immune systems and immunosuppressed people.

**Table 1:** Examples of dietary supplements used for weight control.

Patent No	Title	Patent’s Country	Patent’s Year	Reference
**Examples of dietary supplements in which plants or their metabolites are used as appetite suppressors**				
JP2023041885A	Bioregulator-containing wheat flour and/or rice flour masterbatch for processed food and method for producing the same	Japan	2023	[151]
CN116058499A	Mediterranean diet fruit and vegetable fat-reducing meal replacement powder and preparation method and application thereof	China	2023	[152]
WO2010054469A1	Appetite-suppressing weight management composition	Worldwide applications	2008	[153]
KR102041036B1	Production Method of Crocetin and Health Supplement for Appetite Suppression Comprising Crocetin as an Active Ingredient	Republic of Korea	2018	[154]
WO2014020344A1	Compounds and their effects on appetite control and insulin sensitivity	Worldwide applications	2012	[155]
CA2778381	Dietary supplements and methods of use	United States	2006	[156]
US20060024388A1	Plant-derived or derivable material with appetite-suppressing activity	United States	2002	[157]
US5945107A	Compositions and methods for weight reduction	United States	1998	[80]
**Examples of dietary supplements in which plants or their metabolites are used as hormones and/or neurotransmitters activators**				
KR20220026635A	Composition for preventing or treating obesity and/or metabolic syndrome comprising narcissoside	Republic of Korea	2020	[158]
KR102511950B1	Dietary supplements for weight loss of pill type	Republic of Korea	2020	[159]
KR102461437B1	Pharmaceutical composition for preventing or treating obesity with garcinia cambogia extract and health functional food with the same	Republic of Korea	2022	[160]
KR102511262B1	A process for the preparation of five-grain bread comprising cheonggukjang and five-grain bread comprising cheonggukjang prepared therefrom	Republic of Korea	2022	[161]
US6759063B2	Methods and compositions for reducing sympathomimetic-induced side effects	United States	2002	[162]
KR102438276B1	Anti-inflammatory and antiobesity composition comprising Sargassum horneri extract and method for preparing the same	Republic of Korea	2022	[163]
**Examples of dietary supplements in which plants or their metabolites interact with the hormones in the digestive system and adipose tissue**				
WO2010053949A1	Phytochemical compositions and methods for activating amp-kinase	Worldwide applications	2009	[164]
WO2017064530A1	Agavaceae extract comprising steroidal saponins to treat or prevent metabolic-disorder-related pathologies	Worldwide applications	2015	[165]
**Examples of dietary supplements in which prebiotics are used for weight control**				
JP2023075270A	Prebiotics for treating disorders associated with disturbed composition or function of the gut microbiome	Japan	2023	[166]
CN113750172A	Weight-reducing composition and application thereof in preparation of weight-reducing product	China	2021	[167]
CN115466687A	Composition for reducing body fat content and body weight and application thereof	China	2021	[168]
**Examples of dietary supplements in which probiotics are used for weight control**				
CN116004472A	Clostridium butyricum for relieving obesity and application thereof	China	2023	[169]
CN114480228A	Probiotics for relieving metabolic syndrome, metabolite formula, and application thereof	China	2022	[170]
CN115300605A	Probiotic powder for resisting obesity and losing weight and application thereof	China	2022	[171]
**Examples of dietary supplements in which symbiotics are used for weight control**				
CN114376235A	Weight-reducing probiotics and prebiotics composition beneficial for controlling in vivo fat and preparation method thereof	China	2022	[172]
WO2023070512A1	Composition of prebiotics and probiotics and use thereof	Worldwide applications	2021	[173]

## 6. Discussion

Bad eating habits have significantly increased the number of overweight and obese people. The desire to have an attractive body and the awareness of the risk of incurring chronic degenerative pathologies constrain many people to intervene and counteract this trend. A low-calorie diet and exercise are helpful approaches to achieving this goal. Unfortunately, they require considerable compliance from the population; therefore, many prefer using pills (supplements or medications) to solve this problem. Supplements are preferred by consumers who believe that products of natural origin, unlike synthetic ones, cannot harm their health. The market offers many supplements capable of achieving this goal through different strategies. Self-prescription and the lack of information about their side effects make supplements risky. In Europe and the US, no safety documentation of use is required before a dietary supplement’s introduction into the market [65]. The Food and Drug Administration (FDA) removes a product from the market only after it has been believed to be unsafe. Furthermore, weight management supplements generally contain more than one bioactive compound, which can act alone or in synergy, making it even more difficult to predict the potential risks related to their intake. Another problem is related to self-prescription, which makes it impossible to establish whether side effects depend on improper dosage and/or on the duration of the administration time.

## 7. Conclusions

Obesity is a noteworthy health issue in industrialized countries. Dietary supplements are an alternative to traditional therapies for weight loss control. This review examined the ability to regulate the appetite, nutrient absorption, lipogenesis, energy expenditure, and lipolysis of the principal bioactive compounds employed in dietary supplements (plant extracts, phytochemicals, prebiotics, probiotics, symbiotics, and postbiotics). Little is reported in the literature regarding the toxicity of the bioactive compounds used for these formulations and almost nothing regarding the toxicity of the supplements for weight control on the market.

## 8. Future Directions

To protect consumers, it would be desirable for common legislation to be drafted worldwide, requiring toxicity studies from manufacturers before authorizing the marketing of supplements. It is hoped that more and more supplements containing postbiotics will be brought to market, as they are easily manageable bioactive compounds for marketing (they are easily stored and transportable) and the safest among those reviewed.

## Data Availability

Not applicable.
